# Tick-borne Relapsing Fever and *Borrelia hermsii*, Los Angeles County, California, USA

**DOI:** 10.3201/eid1507.090223

**Published:** 2009-07

**Authors:** Tom G. Schwan, Sandra J. Raffel, Merry E. Schrumpf, Larry S. Webster, Adriana R. Marques, Robyn Spano, Michael Rood, Joe Burns, Renjie Hu

**Affiliations:** National Institute of Allergy and Infectious Diseases, Hamilton, Montana, USA (T.G. Schwan, S.J. Raffel, M.E. Schrumpf); Mt. Wilson Observatory, Mt. Wilson, California, USA (L.S. Webster); National Institute of Allergy and Infectious Diseases, Bethesda, Maryland, USA (A.R. Marques); Los Angeles County Department of Public Health, Baldwin Park, California (R. Spano, M. Rood); California Department of Public Health, Ontario, California (J. Burns, R. Hu)

**Keywords:** Ticks, Borrelia hermsii, bacteria, relapsing fever, vector-borne infections, spirochetes, Los Angeles, USA, research

## Abstract

The primary cause of tick-borne relapsing fever in western North America is *Borrelia hermsii*, a rodent-associated spirochete transmitted by the fast-feeding soft tick *Ornithodoros hermsi*. We describe a patient who had an illness consistent with relapsing fever after exposure in the mountains near Los Angeles, California, USA. The patient’s convalescent-phase serum was seropositive for *B. hermsii* but negative for several other vector-borne bacterial pathogens. Investigations at the exposure site showed the presence of *O. hermsi* ticks infected with *B. hermsii* and the presence of rodents that were seropositive for the spirochete. We determined that this tick-borne disease is endemic to the San Gabriel Mountains near the greater Los Angeles metropolitan area.

## Abstract 2

A patient presumably acquired this illness after exposure to ticks in mountains near Los Angeles.

Tick-borne relapsing fever was first observed in California, USA, in 1921 when 2 persons were infected in a cabin in Nevada County, north of Lake Tahoe (*1*). Another 251 cases of relapsing fever were reported in this state through 1941. Most persons who became ill had exposures at high elevations in various mountain locations (*2*). During this time, tick-borne relapsing fever was found to be endemic near Big Bear Lake in the San Bernardino Mountains, San Bernardino County, in southern California (*2*,*3*). The first human case in the Big Bear Lake area was reported in 1930 (*3*); for the next 12 years, more people became ill with relapsing fever there than in any other single area in the state (*2*).

The initial human cases of relapsing fever in California preceded identification of the vector, which was discovered to be a previously unidentified tick subsequently named *Ornithodoros hermsi* (*4*,*5*). Naturally infected ticks that were collected near Big Bear Lake and Lake Tahoe transmitted spirochetes in the laboratory when the ticks fed on monkeys, mice, and a human volunteer; this transmission showed the role of *O. hermsi* ticks as vectors (*4*,*6*). In 1942, Davis named the *O. hermsi* tick-associated spirochete *Spirochaeta hermsi* (*7*), now recognized as *Borrelia hermsii* (*8*).

Early investigations of relapsing fever associated with *O. hermsi* ticks at Big Bear Lake and Lake Tahoe were seminal for defining the epidemiologic parameters that maintain the enzootic foci in these locations and in other appropriate habitats throughout western North America (*9*). However, since the early field and laboratory studies performed in the 1930s, little work has been conducted to further elucidate the distribution of *B. hermsii* in southern California. In 1989, a total of 6 persons were infected sequentially while staying at different times in the same cabin near Big Bear Lake (*10*), and a year later, 2 persons were diagnosed with relapsing fever when they were hospitalized in Santa Monica, California, after a weekend visit to Big Bear Lake (*11*). During these outbreaks, ticks were not collected and spirochetes were not isolated from the patients’ blood. The most recent review of relapsing fever that included California reported 95 cases for 1978–1998 (*12*). During this 21-year period, 16 (17%) persons were infected in San Bernardino County, but no specific locations were provided.

Information regarding the presence of an enzootic focus of relapsing fever in Los Angeles County, California, is scant and old. A review published in 2002 showed no cases of relapsing fever originating in Los Angeles County during the 20th century (*13*), and a monograph on California ticks had no record of *O. hermsi* ticks being found there (*14*). In 1942, Beck reported 2 cases for 1936–1940 and noted that *O. hermsi* ticks were found in a “nest in garage” at an elevation of 2,134 m (7,000 feet) (*2*). However, no additional information was provided, and we are unaware of any reports that substantiate the presence of human relapsing fever, *O. hermsi* ticks, or *B. hermsii* spirochetes near the Los Angeles metropolitan area. The California State and Los Angeles County health statistics units had no reports of relapsing fever having ever originated in Los Angeles County.

On September 7, 2006, a 51-year-old man cleaned a room associated with a 100-year-old solar telescope at Mt. Wilson Observatory, located at 1,737 m (5,700 feet) elevation in the San Gabriel Mountains, Los Angeles County, in southern California. Numerous rodent nests were present in old cardboard boxes in the room, and the floor was littered with rodent feces, acorn husks, and other debris suggesting many years of rodent occupation. On September 9, the patient carried several cardboard boxes containing rodent nests, and some of the material transferred to his clothing. Later that evening, he noticed 2 “insect bites,” 1 on each leg, just above the sock line. The bites were asymptomatic erythematous macular lesions that enlarged from 2 mm to 6 mm in diameter over the next few days and faded after a week.

On the evening of September 17, the patient experienced a sudden onset of weakness, fever to 38.9°C, shaking chills, and joint and muscle pain; nausea and vomiting developed the next day. On September 20, as the illness persisted, he sought help at a clinic and was prescribed antiemetics, but showed no improvement. On September 21, as the illness worsened, he sought help at a hospital emergency department. Physical examination indicated dehydration and fever (38.9°C). A complete blood count showed mild thrombocytopenia and increased granulocyte count (Table 1). He was treated with ketorolac, metoclopramide, and intravenous saline for possible viral illness and was released. The patient improved over the next several days, but his illness relapsed on September 27, when he had increased weakness, arthralgias and myalgias, fever, shaking chills, and renewed nausea and vomiting. The patient’s illness peaked on September 28 and improved on September 29, but his condition again relapsed on October 1. The patient returned to the emergency department and was hospitalized. Physical examination indicated a temperature of 39.1°C. He was treated with intravenous fluids, antiemetics, and piperacillin/tazobactam. Complete blood counts showed leukocytosis, left shift, and thrombocytopenia (Table 1). Routine blood cultures, which do not support borrelia growth, were negative. He improved a few hours after receiving antimicrobial drug treatment but was mildly hypotensive for a day. The antimicrobial drug was discontinued on October 3, and the patient was again released. Night sweats continued for 2 days after discharge, and he felt generalized weakness for the next several days. He returned to work full time on October 16.

**Table 1:** Laboratory test results for 4 dates during illness of patient presumably ill with tick-borne relapsing fever, Los Angeles County, California, USA, 2006

Test	Sep 21	Oct 1	Oct 2	Oct 3	Reference range
Hematocrit, %	43.3	42.3	33.7	33.3	39–55
Erythrocyte count, 10^–6^/mm^3^	4.6	4.6	3.6	3.6	4.3–5.9
Platelet count/mm^3^	114	145	127	162	130–450
Leukocyte count/mm^3^	8.8	11.5	12.8	9.5	4.8–10.8
Neutrophils, %	83	86	64	58	50–70
Band cells, %		5	11	5	0–4
Urea nitrogen, mg/dL	16	10	11	9	6–20
Creatinine, mg/dL	1.2	1.1	1.1	1.0	0.5–1.2
Aspartate aminotransferase, U/L		32	25	29	10–42
Alanine aminotransferase, U/L		68	52	55	10–60
Total bilirubin, mg/dL		2.2	1.5	1.2	0–1.5
Alkaline phosphatase, U/L		206	143	140	42–121
Sodium, mg/dL	134	133	138	140	135–145
Potassium, mg/dL	3.1	3.6	4.5	4.3	3.6–5.0
Glucose, mg/dL	133	112	119	107	70–110
Albumin, g/dL		2.7	2.1	2	3.2–5.5

## Methods

### Site Investigation

Because of a suspicion that the patient had contracted tick-borne relapsing fever, the exposure site was investigated for ticks. A member of our research team (L.S.W.) designed a novel tick trap in which a white terry cloth towel was wrapped around small blocks of dry ice, which emits CO_2_ and attracts ticks (*15*). Each trap was taped to the end of a 1-m stick and placed in recesses of the room where the patient was potentially exposed to ticks; the traps were left overnight and checked the next morning. During several nights in late October 2006, the traps collected 6 ticks, which were sent to the Rocky Mountain Laboratories, where they were identified as *O. hermsi* nymphs and tested for spirochete infection.

During the nights of June 12–14, 2007, and May 15, 2008, multiple dry ice traps were set again at the observatory in several buildings with abundant signs of rodent activity, including the room that provided the previous ticks. One *O. hermsi* tick was collected in 2007, and 2 nymphs were collected in 2008, all from the previously infested room where the patient had presumably been bitten.

On July 16 and October 23–24, 2008, Tomahawk (Tomahawk Live Trap Co., Tomahawk, WI, USA) and Sherman (H.B. Sherman Traps, Tallahassee, FL, USA) live traps were set on the observatory grounds to capture rodents for serologic testing for antibodies against *B. hermsii*. Whole blood samples were collected from 22 rodents, and the serum samples were tested by immunoblot with whole cell lysates of *B. hermsii* and purified recombinant glycerophosphodiester phosphodiesterase Q (GlpQ), an immunodominant protein in relapsing fever spirochetes but absent from Lyme disease spirochetes (*16*).

### DNA Amplification by PCR and Sequence Analysis of Spirochetes

DNA was extracted from the 6 ticks collected in October 2006 and examined by PCR and DNA sequence analysis of the 16S rRNA, flagellar B protein (*flaB*), gyrase B (*gyrB*), *glpQ*, and variable tick protein (*vtp*) genes and the noncoding intergenic spacer (IGS) locus of *B. hermsii* by using methods described (*17*–*19*).

### Tick Feeding and Spirochete Isolation

The 3 *O. hermsi* nymphs collected on June 14, 2007, and May 15, 2008, each fed on a laboratory mouse (*Mus musculus*). On days 3–9 after feeding, the mice had ruffed hair, and blood collected from the tail vein showed numerous spirochetes per field in a wet preparation viewed by dark-field microscopy at 400×. Each mouse was anesthetized, bled by intracardiac puncture, and euthanized, and 0.2 mL of the blood was passed intraperitoneally into another mouse. The mice were moribund 2 days later and had extremely high spirochetemias. The mice were euthanized immediately after intracardiac puncture, and the blood samples were inoculated into Barbour-Stonner-Kelly (BSK)–H medium (Sigma-Aldrich Inc., St. Louis, MO, USA) with 12% rabbit serum for spirochete isolation. Thin smears of the infected blood were made on glass microscope slides and fixed with methanol to test spirochete reactivity with monoclonal antibody H9724, which is specific for all *Borrelia* spp. (*20*), and monoclonal antibody H9826, which is specific for *B. hermsii* (*21*). DNA was purified from the spirochete cultures for PCR and DNA sequence analysis, and aliquots of the cultures were frozen at –80°C. Our work with the animals was reviewed and approved by the Rocky Mountain Laboratories Animal Care and Use Committee (protocol no. 2006–10).

### Serologic Tests

A convalescent blood sample was drawn from the patient on March 17, 2008, after he signed the informed consent under a protocol approved by the National Institute of Allergy and Infectious Diseases, National Institutes of Health, Institutional Review Board. The serum was tested by the indirect immunofluorescent antibody (IFA) assay with spirochetes fixed on glass microscope slides, including *B. hermsii* originating from the exposure site, *B. hermsii* DAH originating from eastern Washington, and the Lyme disease spirochete *Borrelia burgdorferi* B31. The serum was also tested with fixed antigen preparations of *Rickettsia rickettsii*, *Coxiella burnetii*, and *Yersinia pestis*, the agents of Rocky Mountain spotted fever, Q fever, and plague, respectively. The serum was tested at 8 serial 2-fold dilutions from 1:16 to 1:2,048, and goat anti-human immunoglobulin G conjugated with fluorescein isothiocyanate (1:100) (Kirkegaard and Perry Laboratories Inc., Gaithersburg, MD, USA) was used as the secondary antibody. IFA titers were determined by viewing with a Nikon (Tokyo, Japan) Eclipse E800 epifluorescence microscope at 600× and oil immersion. Serum was also examined at 1:100 dilution by immunoblot with whole cell lysates of the same spirochete panel and with recombinant GlpQ purified by nickel chromatography, as described (*16*,*22*).

## Results

The patient’s clinical history and the ecologic setting of his exposure led us to suspect that he had contracted tick-borne relapsing fever, and we initiated a search for the tick vector. Six *O. hermsi* tick nymphs were collected with the dry ice traps and pooled for molecular analysis to determine presence of spirochete infection. DNA was purified from the pooled ticks, and PCR produced amplicons of the appropriate size for all loci examined. DNA sequences were determined for the 16S rRNA, *flaB*, *gyrB*, *glpQ*, *vtp*, and IGS loci, which identified the Mt. Wilson spirochete (designated MTW-1) as *B. hermsii*, belonging to genomic group II (GG II) (*18*) (GenBank accession nos. EU194839, EU194840, EU194841, EU194842, EU203147, and EU203149). The 16S rRNA, *glpQ*, and IGS sequences were unique from all other sequences for these loci in our *B. hermsii* isolate collection. These unique sequences showed that the PCR products resulted from infected ticks, not from laboratory contamination. The *vtp* sequence was identical to other Type 6 sequences found in genomic group I (GG I) spirochetes.

**Figure 1:**
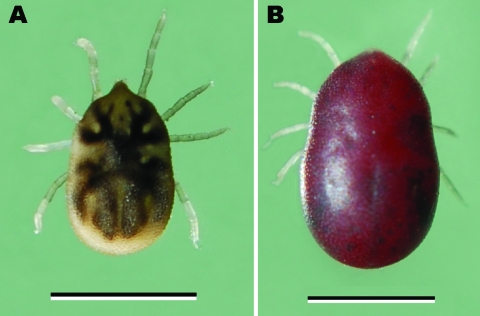
*Ornithodoros hermsi* nymphal tick from Mt. Wilson, California, USA. Panel A shows the nymph before its infective blood meal; panel B shows it after feeding. Scale bars = 2 mm.

**Figure 2:**
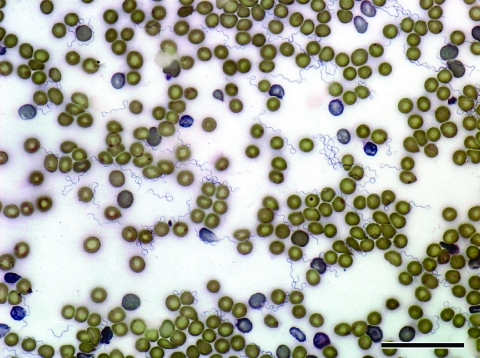
*Borrelia hermsii* MTW-2 in mouse blood (Wright-Giemsa stain) viewed at 600× oil immersion. Scale bar = 40 μm.

The 3 nymphal *O. hermsi* ticks collected on June 14, 2007, and May 15, 2008, each fed on mice (Figure 1) and transmitted spirochetes that produced high spirochetemias (Figure 2). These spirochetes bound both monoclonal antibodies, which identified the spirochetes as *B. hermsii* (data not shown). Spirochetes grew to high cell density in BSK-H medium, and DNA was purified from the second–passage, 100-mL cultures (*23*) and designated MTW-2, MTW-3, and MTW-4. DNA samples purified from the cultures were subjected to PCR, and DNA sequences of the amplicons were determined for the same 6 loci (GenBank accession nos. EU194843, EU194845, EU194846, EU194847, EU203148, and EU203150). All sequences determined for each locus were identical for the 3 isolates. Sequences for the 16S rRNA, *flaB*, and *gyrB* genes and for the IGS locus were identical to those sequences for MTW-1. This similarity identified MTW-2, MTW-3, and MTW-4 as *B. hermsii* belonging to GG II. The *glpQ* sequences for MTW-1 and for MTW-2, MTW-3, and MTW-4 contained 2 synonymous base differences (99.8% identity). However, the *vtp* DNA sequence for MTW-2, MTW-3, and MTW-4 were identical and unique from all other *vtp* sequences in the database (*18*), although the sequences showed similarities with other Vtp type 5 sequences with 94.6% identity (*18*). The *vtp* sequences in MTW-1 and MTW-2 represented different Vtp types (type 6 and 5, respectively) and shared only 78.5% DNA and 69.3% amino acid identities.

**Figure 3:**
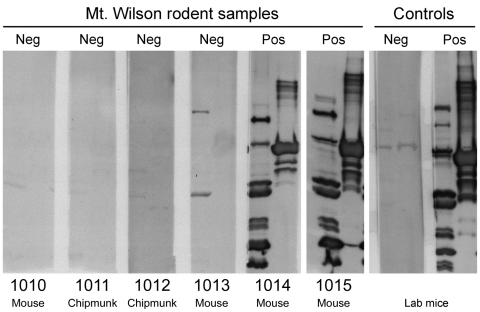
Immunoblot analysis of serum samples from brush mice (*Peromyscus boylii*) and Merriam chipmunks (*Tamias merriami*) captured at Mt. Wilson Observatory in California, USA. Each sample was tested at a dilution of 1:100 with a whole-cell lysate of *Borrelia hermsii* MTW-2 isolated from Mt. Wilson (left lane of each membrane) and purified recombinant GlpQ (right lane of each membrane). Results and animal numbers are presented above and below each panel, respectively. Neg, negative; pos, positive.

Setting the traps resulted in capturing 22 small mammals from 4 species: 14 California ground squirrels (*Spermophilus beecheyi*), 2 Merriam chipmunks (*Tamias merriami*), 5 brush mice (*Peromyscus boylii*), and 1 California mouse (*Peromyscus californicus*). Immunoblot analysis of serum samples from the 22 animals showed that 2 of the brush mice were strongly reactive with antibody reactivities equal to those of the positive control sample (Figure 3). The other 20 samples were negative. The positive serum samples contained antibodies that bound to ≥9 proteins in the *B. hermsii* whole-cell lysates and to the recombinant purified GlpQ. The 2 seropositive mice were captured in traps set against the exterior walls of the building in which the patient was presumed to have been infected.

The patient’s convalescent-phase serum sample was seropositive for relapsing fever with the highest IFA titer (2,048) to *B. hermsii* MTW-2 originating from Mt. Wilson and with the lowest titers (512 and 128) to *B. hermsii* DAH from eastern Washington and Lyme disease spirochete *B. burgdorferi*, respectively (Table 2). IFA titers were negative with the other bacterial antigens (Table 2). Immunoblots were also positive for antibodies against GlpQ, and the serum samples had the strongest reactivity to the Mt. Wilson spirochete (data not shown).

**Table 2:** Patient convalescent-phase IFA assay titers to *Borrelia hermsii* and other vector-borne bacterial pathogens, Los Angeles County, California, USA, 2006*

Species	IFA titer
*B. hermsii* MTW-2	2,048
*B. hermsii* DAH	512
*B. burgdorferi* B31	128
*Rickettsia rickettsii*	<16
*Coxiella burnetii*	<16
*Yersinia pestis*	<16

*IFA, indirect immunofluorescent antibody.

## Discussion

The diagnosis of relapsing fever was not considered during the patient’s illness, and his blood was not examined for spirochetes during his acute-phase episodes. Thus, we were unable to isolate spirochetes from his blood or obtain an acute-phase serum sample to compare with the convalescent-phase sample. However, we conclude that the patient had contracted relapsing fever on the basis of several factors: his clinical history and exposure consistent for this disease, his recovery with antimicrobial drug treatment, the high IFA titer to *B. hermsii*, the positive immunoblot, the presence of *O. hermsi* ticks in the room of exposure, the presence of *B. hermsii* in the tick pool, and the isolation of spirochetes that originated from 3 naturally infected ticks. Additionally, the patient lived on Mt. Wilson and had not traveled recently to other known locations where this disease is endemic. Cumulatively, these findings demonstrate an enzootic focus of tick-borne relapsing fever in Los Angeles County at a location only 10 km (6.5 miles) from downtown Pasadena.

A few vertebrate animals potentially involved with this focus were investigated. Douglas tree squirrels (*Tamiasciurus douglasii*), which are important hosts for *O. hermsi* ticks and *B. hermsii* spirochetes, inhabit the Sierra Nevada Mountains to the north but are absent from the San Gabriel and San Bernardino Mountains (*9*,*24*,*25*). Other sciurids, including the California ground squirrels (*S. beecheyi*), Western gray squirrels (*Sciurus griseus*), and Merriam chipmunks (*T. merriami*), are abundant on the observatory grounds. Several species of mice (*Peromyscus* spp.) are also found at the observatory. In 1947, Longanecker collected 90 *O. hermsi* ticks from an active deer mouse nest near Big Bear Lake (*26*). Many larval and nymphal ticks had recently fed, and some specimens were infected with spirochetes. Our serologic results suggest that brush mice (*P. boylii*) are involved in the enzootic focus on Mt. Wilson.

Western bluebirds (*Sialia mexicana*) are also abundant around the observatory and nest in tree hole cavities. *O. hermsi* ticks have been found in bluebird nests in the Sierra Nevada Mountains and in British Columbia, Canada (*26*,*27*); these birds may also serve as hosts for the ticks in this site. Further work is needed to determine the role that these and other mammals and birds may play in maintaining the ticks and spirochetes in the mountains of southern California.

The investigation provided several results concerning the distribution and genetic diversity of *B. hermsii*. First, spirochetes from all ticks from Mt. Wilson belonged to GGII, extending south by ≈900 km (560 miles), the known geographic distribution of *B. hermsii* in this genomic group (*19*). Therefore, spirochetes in both genomic groups are likely to occur throughout the north-to-south range of these organisms in far western North America. Second, GGII spirochetes were found in naturally infected *O. hermsi* ticks; all GGII isolates examined previously came from blood of clinically ill patients (*19*). Third, the Mt. Wilson spirochetes MTW-1 and MTW-2 had identical or nearly identical DNA sequences at all loci examined except for the variable tick protein gene (*vtp*). The antigenically diverse Vtp sequences occurring in ticks in the same location may allow spirochetes of a specific Vtp type to reinfect previously infected hosts that are possibly immune to spirochetes of another Vtp type (*18*).

Our study found an enzootic focus of the relapsing fever spirochete *B. hermsii* and its tick vector *O. hermsi* on Mt. Wilson near the Los Angeles metropolitan area. The patient in our study probably contracted relapsing fever there, although he was not tested for this infection during his illness. During the third acute-phase episode, he was hospitalized; hospitalization occurs frequently among patients if this disease is not diagnosed early. A retrospective analysis of relapsing fever cases in California and Washington for 1995–2005 showed that 46% and 80% of cases in each state, respectively, required hospitalization (*28*). Therefore, we emphasize that physicians and other healthcare providers should consider relapsing fever as a possible diagnosis when patients seek treatment for an acute, febrile, recurrent disease after being exposed in the mountains of southern California and other regions where relapsing fever is endemic. We also recommend collection of blood samples during acute episodes for subsequent analysis and possible retrospective clinical testing and diagnosis.
